# Primary coenzyme Q10 deficiency due to *COQ8A* gene mutations

**DOI:** 10.1002/mgg3.1420

**Published:** 2020-08-02

**Authors:** Linwei Zhang, Tetsuo Ashizawa, Dantao Peng

**Affiliations:** ^1^ Department of Neurology China‐Japan Friendship Hospital Beijing China; ^2^ Houston Methodist Research Institute and Department of Neurology Houston Methodist Neurological Institute Houston Texas USA

**Keywords:** coenzyme Q10, *COQ8A* gene, deficiency, mutations

## Abstract

**Background:**

Primary deficiency of coenzyme Q10 deficiency‐4 (COQ10D4) is an autosomal recessive cerebellar ataxia with mitochondrial respiratory chain disfunction. The main clinical manifestation involves early‐onset exercise intolerance, progressive cerebellar ataxia, and movement disorders. *COQ8A* gene mutations are responsible for this disease. Here, we provide clinical, laboratory, and genetic findings of a patient with cerebellar ataxia caused by compound heterozygous mutations in *COQ8A* gene.

**Methods:**

A male patient from a non‐consanguineous Chinese family underwent detailed physical and auxiliary examination. After exclusion of acquired causes of ataxia, Friedreich's Ataxia, and common types of spinocerebellar ataxia, the patient was subjected to whole exome sequencing (WES) followed by confirmation of sequence variants using Sanger sequencing. His asymptomatic parents, two brothers and one sister were genotyped for these variants.

**Results:**

This patient showed early‐onset exercise intolerance and progressive cerebellar ataxia, wide‐based gait and tremor, accompanied by symptoms of dysautonomia. His serum lactate level was elevated and plasma total Coenzyme Q10 (CoQ10) was decreased. Brain MRI showed cerebellar atrophy, and X‐ray of the spine revealed thoraco‐lumbar scoliosis. Compound heterozygous mutations in the *COQ8A* gene were identified through WES: c.1844_1845insG, p.Ser616Leufs*114 and c.902G>A, p.Arg301Gln. After treatment with ubidecarenone, 40 mg three times per day for 2 years, the symptoms dramatically improved.

**Conclusions:**

We identified a patient with COQ10D4 caused by novel *COQ8A* mutations. Our findings widen the spectrum of *COQ8A* gene mutations and clinical manifestations.

## Graphical abstract

We identified a patient with COQ10D4 caused by novel COQ8A mutations through WES. Our findings widen the spectrum of COQ8A gene mutations and clinical manifestations.

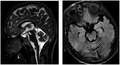

## 1 INTRODUCTION

Primary coenzyme Q10 (CoQ10) deficiency is a group of autosomal recessive cerebellar ataxias with defective mitochondrial respiration caused by multiple genetic mutations. The phenotype of primary CoQ10 deficiency is characterized by early‐onset exercise intolerance, progressive cerebellar ataxia, intellectual disability, seizure, stroke‐like episodes, mitochondrial myopathy, hypogonadism, and steroid‐resistant nephrotic syndrome, with the age at onset ranging from infancy to late adulthood (Alcazar‐Fabra, Trevisson, & Brea‐Calvo, 2018; Gironi et al., 2004; Horvath et al., 2006; Mollet et al., 2008). Primary CoQ10 deficiency‐4 (COQ10D4) is the most frequent form of primary CoQ10 deficiency and caused by mutations of *COQ8A* (OMIM*606980) gene (also known as *ADCK3* or *CABC1*). *COQ8A* encodes the homolog of yeast coq8 (Lagier‐Tourenne et al., 2008), plays an important role in CoQ10 biosynthesis and ATP production, and possesses ATPase activity (Lagier‐Tourenne et al., 2008; Reidenbach et al., 2018). CoQ10 (also known as ubiquinone) acts as an electron carrier in the mitochondrial respiratory chain and plays a role as an antioxidant and membrane stabilizer (Yubero et al., 2018).

Here, we report the clinical, biochemical, and genetic investigation of a patient from a non‐consanguineous family with an autosomal recessive cerebellar ataxia due to novel compound heterozygous mutations in the *COQ8A* gene. This patient exhibited adolescent onset exercise intolerance, progressive cerebellar ataxia, tremor, and dysautonomia. After oral supplement with ubidecarenone 40 mg, three times per day for 2 years, the patient's neurological symptoms were significantly ameliorated.

## 2 PATIENT AND METHODS

### 2.1 Ethical approval

The study was conducted in accordance with the declaration of Helsinki and was approved by the ethics committee of China‐Japan Friendship Hospital. All participants provided written informed consent.

### 2.2 Case presentation

**Figure 1:**
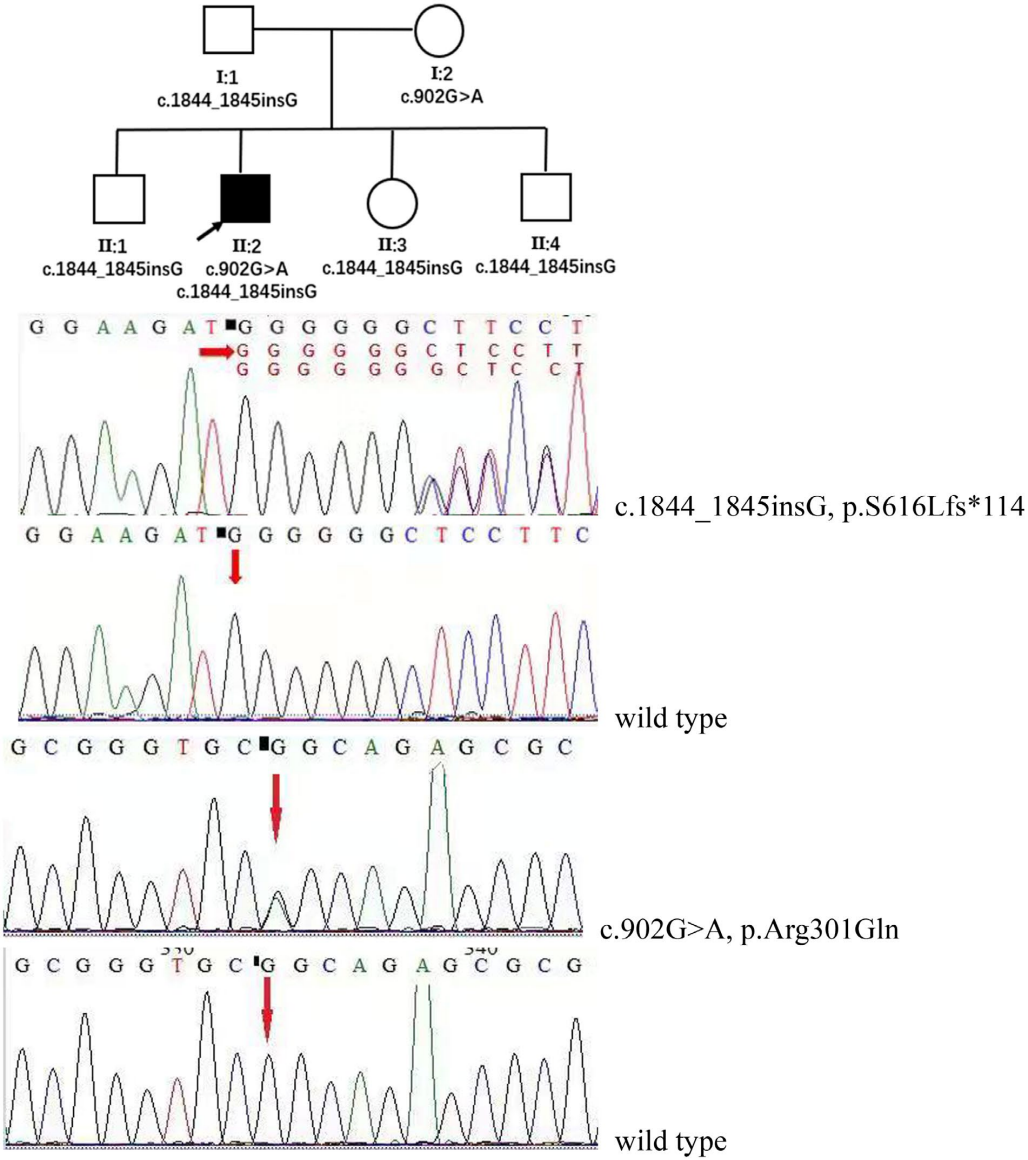
Pedigree and Sanger sequencing validation of compound heterozygous mutations c.1844_1845insG, p.Ser616Leufs*114 & c.902G>A, p.Arg301Gln of *COQ8A* gene in this pedigree. (clear square, male; clear circle, female; black square, index patient)

The index Patient (Ⅱ:2, pedigree showed in Figure 1) is a 35‐year‐old male who was born to healthy non‐consanguineous parents and had three asymptomatic siblings. In his 9‐years old he suffered exercise intolerance due to notable muscle fatigue. He started to experience unsteady walking, with involuntary jerking and tremor of the head at 13 years of age. His gait gradually worsened to obvious wide‐based staggering gait with the development of slurred speech and clumsy hands. He also complained of memory loss, which was treated with sodium valproate, clonazepam, and atenolol with slight improvement. The patient suffered from chronic constipation, occasional urinary incontinence, and erectile dysfunction since he was 16 years old.

His past medical history is significant for successfully treated childhood tuberculosis. There is no family history of similar neurological disorders.

Neurological examination revealed mild dysarthria, overt head tremor, bilateral dysmetria, and intention tremor on nose‐finger and heel‐shin tests, and wide‐based ataxic gait with inability to walk in tandem. SARA score was 13 (gait 3, stance 3, sitting 0, speech 1, finger chase 1, nose‐finger test 2, fast alternating hand movements 1, and heel‐shin slide 2). Kayser–Fleischer Rings were absent, and vision and hearing were normal. Motor and sensory examination and deep tendon reflexes were normal. There was no Babinski sign or other pathological reflexes. The Wechsler intelligence test was normal, and self‐rating depression scale showed mild depression.

His serum lactate levels were elevated, and plasma CoQ10 concentrations were decreased. The remaining blood tests, including liver function, serum creatine kinase (CK), autoimmune antibodies, thyroid function, blood smear for acanthocytosis, and plasma levels of vitamins (B1, B2, B6, B9, B12, A, D, E), copper and ceruloplasmin were all normal. Cerebrospinal fluid (CSF) was normal including inflammatory, immunological, and infectious indices. Electromyography (EMG), nerve conduction velocity (NCV), and brainstem auditory evoked potential (BAEP) were normal. Initial DNA analyses using capillary electrophoresis of PCR products excluded FRDA and SCA 1, 2, 3, 6, 7, 8, 10, 12, 17 and DRPLA. The patient declined muscle biopsy.

### 2.3 Whole exome sequencing

DNA was extracted from peripheral leukocytes of the patient and all available family members according to the standard protocol and signed informed consent as approved by the China‐Japan Friendship Hospital. Genomic DNA of the proband was subjected to WES using the Ion Torrent AmpliSeq Exome RDY kit (BGI Tech, Hong Kong). Variant call files were analyzed with Ingenuity Variant Analysis (Qiagen, Redwood City, CA) using an autosomal recessive model. Clean reads were aligned on the human assembly GRCh37 (as known as hg19) by BWA. Small insertions/deletions (INDELs) and single nucleotide variants (SNVs) were called by GATK and annotated by ANNOVAR. Several filtration steps to obtain putative pathogenic variants were processed. The functional effects of protein variants were predicted by SIFT, PolyPhen2, and MutationTaster. Disease association databases (e.g., HGMD, OMIM, and ClinVar) and genetic variation databases (e.g., 1000 Genomes Project, ESP6500, and ExAC) were used in the filtering process as well. Potential pathogenic variants were validated by conventional Sanger sequencing, and his family members were included for segregation analysis.

### 2.4 Quantification of lactate and CoQ10 levels

Lactic acid of plasma was elevated to 6.9 mmol/L at rest (normal range: 0.5–1.6 mmol/L). The plasma level of total CoQ10, detected by high‐performance liquid chromatography (HPLC), was 0.59 µg/ml (normal range: 1.31 ± 0.38 µg/ml (Zhang, Gu, Wang, Chen, & Zhang, 2016)).

### 2.5 Imaging

**Figure 2:**
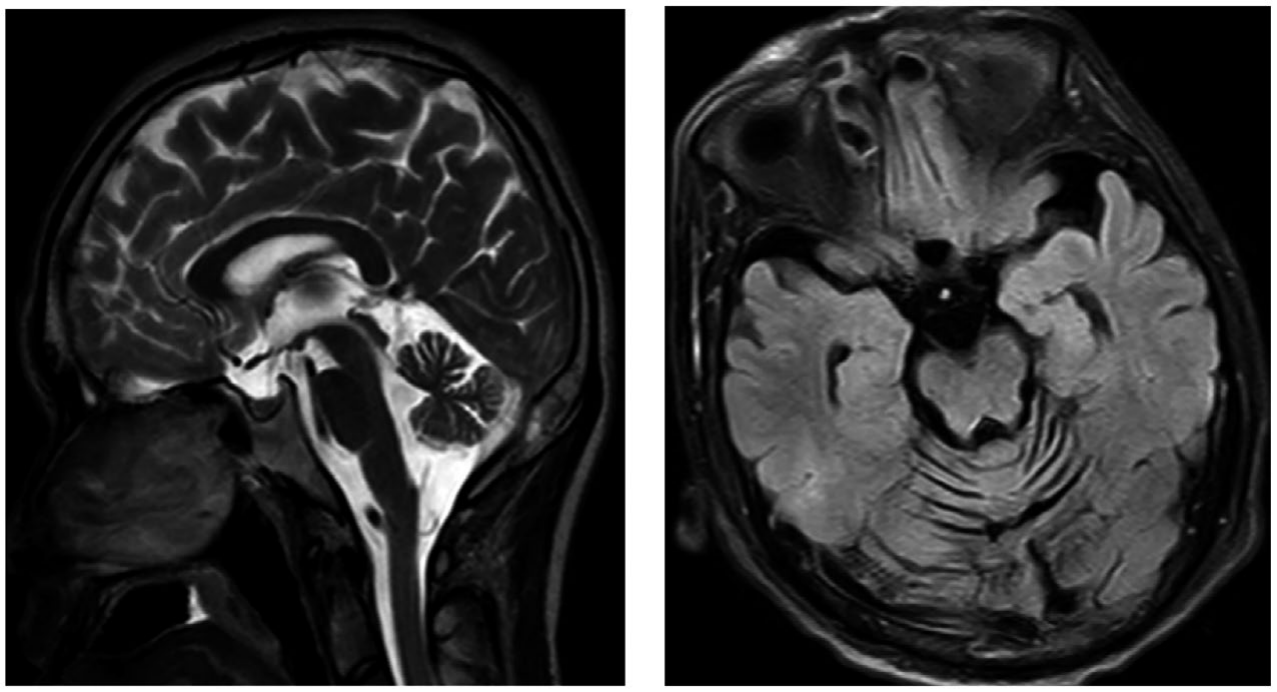
Brain MRI show cerebellar atrophy of the index patient(Ⅱ:2). Left: Sagittal scan, Right: axial scan

**Figure 3:**
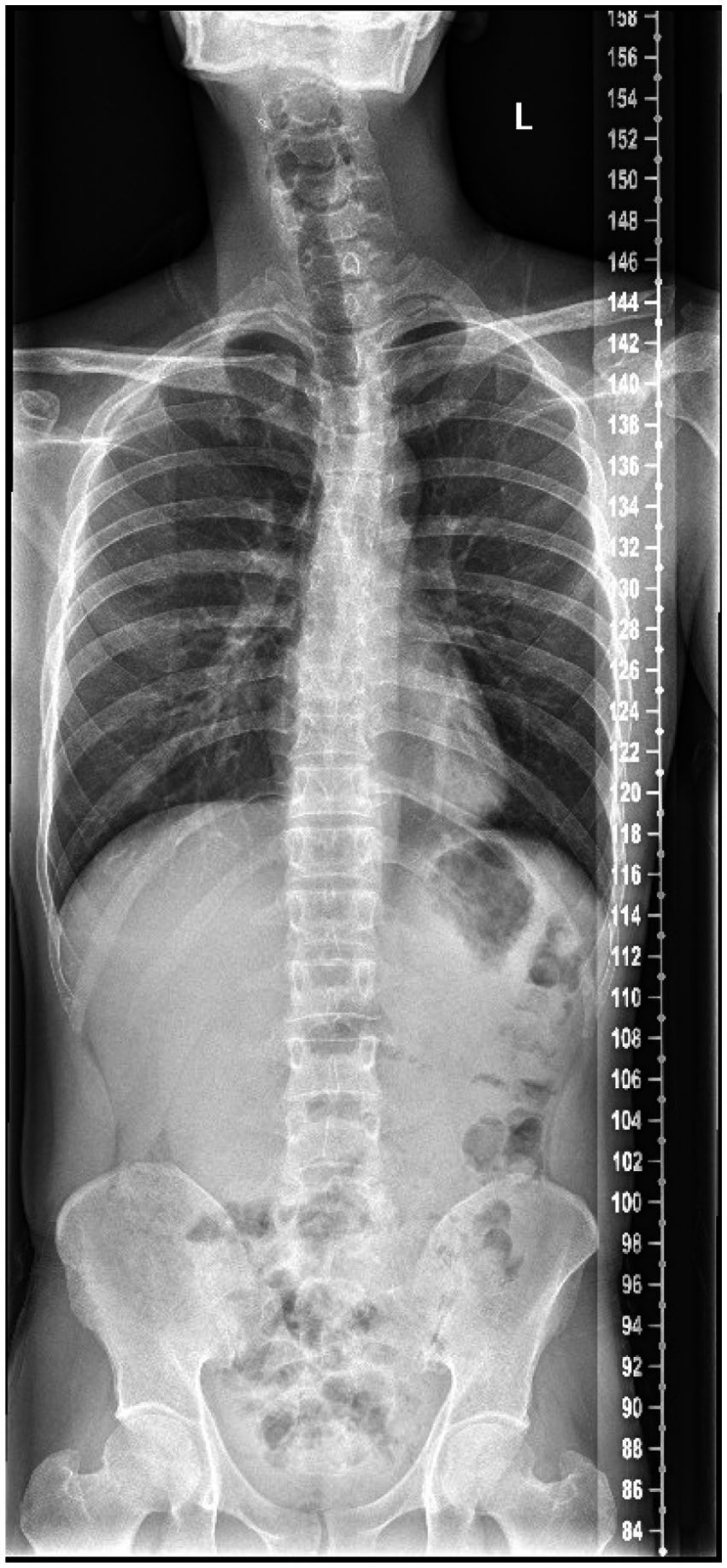
X ray of the spine show thoraco‐lumbar scoliosis of the index patient(Ⅱ:2)

Brain MRI showed remarkable symmetric cerebellar atrophy (Figure 2). X‐ray of the spine showed thoraco‐lumbar scoliosis (Figure 3).

### 2.6 WES and variants validation

We used the transcript sequence (NM_020247.5) of the *COQ8A* gene and discovered compound heterozygous variants in *COQ8A* (c.902G>A, p.Arg301Gln and c.1844_1845insG, p.Ser616Leufs*114) in the proband. Sanger sequencing confirmed these results with each parent as a heterozygous carrier for one of the mutations (Figure 1) and the proband's siblings (II:1, II:3, and II:4) were c.1844_1845insG carriers (Figure 1). The c.1844_1845insG mutation was pathogenic and described before (Liu et al., 2014), this frameshift mutation created a premature stop codon and replacement of the last 32 amino acids of the *COQ8A* protein by 113 incorrect amino acids (Liu et al., 2014). c.902G>A, p.Arg301Gln was a novel missense mutation, located adjacent to a reported mutation c.901C>T (p.Arg301Trp) (Chang et al., 2018; Sun et al., 2019; Traschütz et al., 2020), and predicted to be harmful (SIFT: damaging, PolyPhen2: possibly damaging, MutationTaster: disease causing). The 301 arginine residue affected by the mutation is highly conserved in evolution.

### 2.7 Treatment and outcome

After we detected the decreased level of plasma total CoQ10, CoQ10 supplementation was immediately started with ubidecarenone of 40 mg three times per day. After 2 weeks of therapy, his self‐reported fatigue and exercise intolerance notably improved. After 2 years of therapy, his ataxia and head tremor diminished. His SARA total score improved from 13 to 8 (gait 1.0, stance 2.0, sitting 0, speech 1.0, finger chase 1.0, nose‐finger test 1.0, fast alternating hand movements 1.0, and heel‐shin slide 1.0). When he stopped ubidecarenone for a month, his condition dramatically deteriorated, rendering him to resume CoQ10 therapy. Constipation and urinary incontinence were also mostly relieved after 2 years of CoQ10 supplementation, but erectile dysfunction still existed.

## 3 DISCUSSION

By WES we identified compound heterozygous mutations of the *COQ8A* gene in a Chinese ARCA family, and we excluded common causes of ataxia. Mutations of *COQ8A* are the most common cause of primary coenzyme Q10 deficiency‐4 (COQ10D4), also known as ARCA2 and SCAR9（Lagier‐Tourenne et al., 2008）. *COQ8A* mutations, first described by Aure et al. (2004) and Mollet et al. (2008), exhibit a mild disease course (Mignot et al., 2013). Several genes encoding CoQ biosynthetic proteins have been shown to have pathogenic variants causing human primary CoQ deficiency, including *PDSS1*,* PDSS2*,* COQ2*,* COQ4*,* COQ5*, COQ6, *COQ7*, *COQ8A*, *COQ8B*, and *COQ9*. While the worldwide frequency of primary Coenzyme Q deficiencies has been estimated as 1/50,000 (Alcazar‐Fabra et al., 2018), it is very rare in China; only 11 patients with primary coenzyme Q deficiency‐7 (COQ10D7; with a founder mutation *COQ4*: c.370G>A, p.(Gly124Ser) have been reported from southern China (Yu et al., 2019). The clinical manifestations of COQ10D4 are also highly heterogeneous, age of onset can range from infant to late adult onset (Galosi et al., 2019; Horvath et al., 2012; Mignot et al., 2013; Traschütz et al., 2020). The usual clinical phenotypes are progressive gait ataxia and movement disorders (dystonia, tremor, chorea, jerk myoclonus) (Liu et al., 2014; Mignot et al., 2013; Traschütz et al., 2020), similar to our patient. Other neurological abnormalities include adolescence onset exercise intolerance due to fatigability, seizures, stroke‐like episodes, intellectual disability, spasticity, ophthalmic involvement, decreased visual acuity, sensorineural hearing loss, depression, and pes cavus have been reported (Alcazar‐Fabra et al., 2018; Aure et al., 2004; Blumkin et al., 2014; Horvath et al., 2012; Lagier‐Tourenne et al., 2008; Mollet et al., 2008; Traschütz et al., 2020). Cognitive impairment is often observed in early‐onset childhood primary coenzyme Q10 deficiency cases (Blumkin et al., 2014), with epileptic encephalopathy. Our patient exhibited relative normal cognitive state. Steroid‐resistant nephrotic syndrome and isolated myopathy were often reported in other types of primary coenzyme Q deficiency (Ashraf et al., 2013; Gempel et al., 2007; Horvath et al., 2006), but not considered as the usual presentation of COQ10D4. Our patient suffered from constipation, urinary incontinence, erectile dysfunction, and other autonomic dysfunctions which have rarely been reported before.

Laboratory tests may detect elevated levels of lactic acid and creatine kinase, and a decreased level of CoQ10 (Gempel et al., 2007; Horvath et al., 2006), our patient had mildly elevated lactate levels, normal CK, and decreased CoQ10 concentration of plasma.

It is unfortunate that the patient declined muscle biopsy since the HPLC assay for the CoQ10 level in skeletal muscle is the golden standard for CoQ10 deficiency. Lymphoblast cells or cultured skin fibroblasts can be alternative tissues for CoQ10 evaluation (Liu et al., 2014; Shalata et al., 2019), although the results of different tissue may not always accurately predict the result of the skeletal muscle (Lagier‐Tourenne et al., 2008; Mignot et al., 2013). We also lack the data of plasma lactic acid and CoQ10 levels after treatment. Low levels of CoQ10 may also be present in secondary CoQ10 deficiency like multiple acyl‐CoA dehydrogenase deficiency (MADD), ataxia with oculomotor apraxia (AOA), and mitochondrial encephalopathies (Horvath, 2012). Skeletal muscle histopathology would have been useful for assessing ragged‐red fibers, SDH‐positive and COX‐deficient fibers, and type 1 myofiber lipid droplets (Gempel et al., 2007; Horvath et al., 2006).

Imaging studies showed marked cerebellar atrophy and thoracic scoliosis. Cerebellar atrophy is the most common radiological sign of COQ10D4 patients while global brain atrophy, stroke‐like lesions, thin corpus callosum, and ventricular enlargement (Horvath et al., 2012; Mignot et al., 2013; Mollet et al., 2008), and thoraco‐lumbar scoliosis have been reported (Horvath et al., 2006; Musumeci et al., 2001).

**Figure 4:**
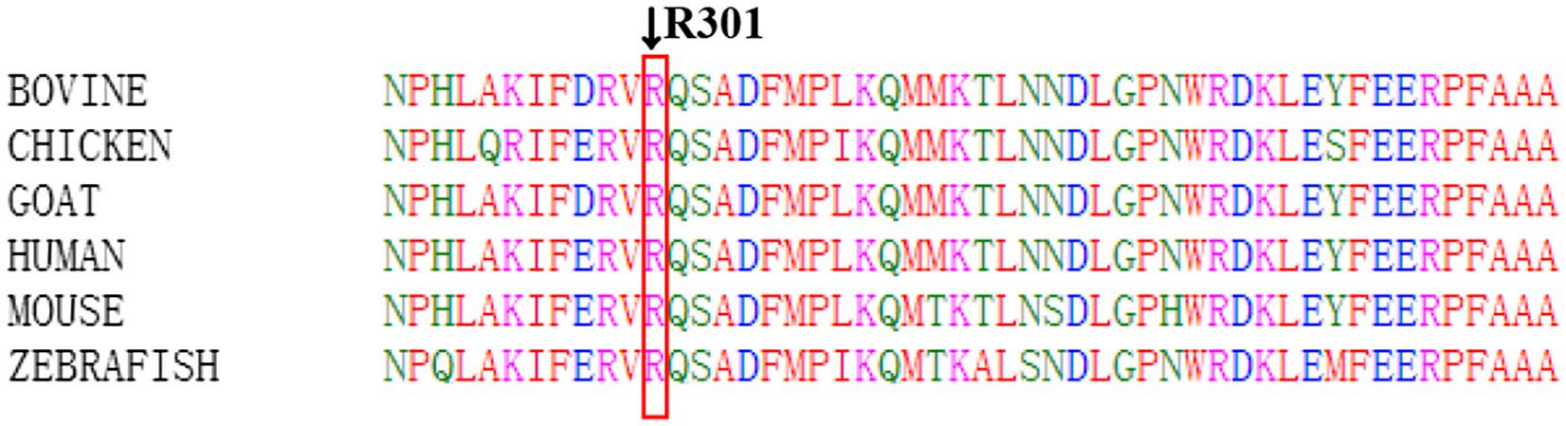
Evaluation of the mutation p.Arg301Gln is highly conserved among species

To date, more than 50 pathogenic mutations of *COQ8A* gene in more than 50 COQ4D patients have been reported (https://www.genecards.org) (Galosi et al., 2019; Traschütz et al., 2020). WES of this patient showed compound heterozygous mutations c.902G>A, p.Arg301Gln and c.1844_1845insG, p.Ser616Leufs*114 in the *COQ8A* gene. The latter variant c.1844_1845insG (p.Ser616Leufs*114) causes a frameshift and has been previously described in a homozygous state in two affected siblings with an adolescent onset of cerebellar ataxia and severe myoclonus from a consanguineous Pakistani family (Liu et al., 2014). In contrast, c.902G>A, p.Arg301Gln is a novel missense mutation at a nucleotide adjacent to a reported mutation c.901C>T (p.Arg301Trp), which was found in Italian and North American patients (Chang et al., 2018; Sun et al., 2019; Traschütz et al., 2020). The c.902G>A is predicted to be disease causing by SIFT, PolyPhen2, and MutationTaster, and causes a change in a highly conserved amino acid residue (R301Q, Figure 4), this mutation could cause GQα3 helix (290‐303AA) disruption (Traschütz et al., 2020).

The additional compelling evidence for the CoQ10 deficiency in our patient comes from clinical responses to CoQ10 supplementation, including the improvement in exercise intolerance and unsteady gait, a response similar to that found in the majority of cases of primary CoQ10 deficiency (Barca et al., 2016; Mignot et al., 2013). CoQ10 supplementation shows such a robust therapeutic efficacy that it has led to alleviation of cognitive impairment, weight loss, and even scoliosis in primary CoQ10 deficiency (Blumkin et al., 2014; Musumeci et al., 2001). Dosage and course of CoQ10 supplement have not been standardized, and results have been variable. The dose of oral CoQ10 (ubiquinone, ubiquinol, idebenone, and ubidecarenone) ranged from 5 mg/kg/day to 3000 mg/day in treatment of CoQ10 deficiencies (Musumeci et al., 2001). Most COQ10D4 patients experienced symptomatic improvement (Chang et al., 2018; Mignot et al., 2013; Jacobsen et al., 2018) although some patients with *COQ8A* mutations showed no satisfactory response (Gerards et al., 2010; Lagier‐Tourenne et al., 2008; Mollet et al., 2008). Early and sustained CoQ10 supplementation appears to be important for a favorable outcome, suggesting that persistent ongoing damage to target tissues and irreversibility of established damages are determinants of therapeutic efficacy (Blumkin et al., 2014). We administered oral ubidecarenone 40 mg, three times a day for 2 weeks to our patient, with an initial subjective improvement of fatigue and exercise intolerance, followed by a remarkable improvement of ataxia and tremor with a substantially lower (by five points) total SARA score. The therapeutic efficacy was further confirmed by a therapeutic challenge of temporarily halting the doses of CoQ10.

The remarkable clinical response of CoQ10 supplement in this patient with *COQ8A* mutations highlights the importance of therapeutic trials of CoQ10 in patients with unknown cause of ataxia. The disease severity may not correlate with serum lactate and CoQ10 levels or mitochondrial morphology in muscle biopsy (Emmanuele et al., 2012; Rahman, Clarke, & Hirano, 2012). So, clinical improvement by CoQ10 supplementation may be the clinical hallmark of primary CoQ10 deficiency.

## 4 CONCLUSION

In summary, our report of the first case of COQ10D4 from China extends the phenotypic and genotypic spectrum of the disease. The striking response to CoQ10 supplementation in our patient highlights the importance of identifying this treatable cause of complex ataxia‐associated syndrome.

## STANDARD PROTOCOL APPROVALS AND PATIENT CONSENTS

The study protocol was reviewed and approved by the Ethics Committee (IRB) of China‐Japan Friendship Hospital. Written informed consent was obtained from the patient and his family for genetic analysis and publication of this research. A copy of the written consent is available for review by the Editor of this journal.

## CONFLICT OF INTEREST

The authors declare that they have no competing interests.

## AUTHOR CONTRIBUTIONS

Dr. Linwei Zhang—acquisition of data, drafting the manuscript, analysis and interpretation of data.

Dr. Tetsuo Ashizawa—critical revision of the manuscript for important intellectual content.

Dr. Dantao Peng—critical revision of the manuscript, study supervision.
